# Cellular and Humoral Responses in Dialysis Patients after Vaccination with the BNT162b2 or mRNA-1273 Vaccines

**DOI:** 10.3390/life13020474

**Published:** 2023-02-08

**Authors:** Ilias Mavrovouniotis, Asimina Fylaktou, Maria Stagou, Konstantinos Ouranos, Georgios Lioulios, Efthimia Evgenikaki, Maria Exindari, Georgia Gioula

**Affiliations:** 1 Microbiology Department, Medical School, Aristotle University of Thessaloniki, 54642 Thessaloniki, Greece; 2 National Peripheral Histocompatibility Center, Immunology Department, Hippokration General Hospital, 54642 Thessaloniki, Greece; 3 Department of Nephrology, School of Medicine, Aristotle University of Thessaloniki, Hippokration Hospital, 54642 Thessaloniki, Greece

**Keywords:** SARS-CoV-2 vaccination, end-stage renal disease, hemodialysis

## Abstract

**Simple Summary:**

Patients with end-stage renal disease (ESRD) requiring hemodialysis (HD) constitute a high-risk group in terms of susceptibility to and severity of infection. High mortality rates have been observed in this population group with SARS-CoV-2 infection, and thus strategies to halt viral transmission are deemed crucial. Vaccination with an mRNA-based vaccine has been shown to decrease the risk of severe disease and substantially reduce mortality, both in healthy individuals and ESRD patients. The cellular and humoral immune responses elicited in HD patients, however, are diminished when compared to healthy controls, with the waning immunity that ensues placing them at high risk for reinfection. Several variables have been found to be associated with this suboptimal response to vaccination, with immunosuppression, older age, and underlying comorbidities playing a central role. Lately, the effects of booster dose administration have been studied in HD patients, with results indicating an enhanced immune response capable of inducing a protective immune profile in this group.

**Abstract:**

The outbreak of SARS-CoV-2 has raised considerable concern about the detrimental effects it can induce in public health, with the interest of the scientific community being focused on the development of preventive and therapeutic approaches. Patients with end-stage renal disease (ESRD) are amongst vulnerable populations for critical illness owing to the presence of other comorbidities, their defective immune system, and their inability of self-isolation. To date, vaccination constitutes the most promising method to manage viral dispersion. Therefore, it is particularly important to investigate the effectiveness of available vaccines against SARS-CoV-2 in this risk group. Here, we summarize initial experience regarding the humoral and cellular immune responses elicited in dialysis patients after completion of the recommended vaccination regimen, as well as after booster dose administration, with one of the two mRNA vaccines, namely, BNT162b2 and mRNA-1273. In conclusion, a significantly diminished and delayed immune pattern was observed in ESRD patients compared to healthy population, with a peak in antibody titers occurring 3–5 weeks after the second dose. A booster dose significantly augmented the immune response in dialysis patients with either mRNA-based vaccine. Variables adversely correlating with the weak immunogenicity observed in dialysis patients include immunosuppressive therapy, older age, comorbidities, longer time in hemodialysis treatment, and higher body mass index. On the contrary, previous COVID-19 infection and administration of the mRNA-1273 vaccine are deemed to induce a more favorable immune response. Further investigation is needed to thoroughly understand the efficacy of mRNA-based vaccines in hemodialysis patients and define predictive factors that can influence it.

## 1. Introduction

The COVID-19 pandemic poses a considerable threat to public health [1], with the World Health Organization recording more than 249 million confirmed cases and 5 million deaths. Control measures to halt virus transmission focus on preventive strategies, with vaccines currently serving as the most crucial means of mitigating viral dispersion. This has resulted in in an acceleration of vaccine development and manufacturing processes [2], with candidate vaccines moving to phase III clinical trials only a few months after the detection of the first COVID-19 case [3]. Candidate vaccines against COVID-19 differ based on the mechanisms exploited to elicit an efficient immune response. Examples include the viral-vector vaccines carrying the DNA-coding spike protein (ChAdOx1 nCoV-19/Oxford-AstraZeneca, Cambridge, England), the traditional platform of inactivated virus (CoronaVac/Sinovac), vaccines based on viral components (NVX-CoV2373/Novavax), and mRNA-based vaccines (BNT162b2/Pfizer-BioNTech, mRNA-1273/Moderna) [4,5]. The latter vaccines utilize host cells to express the corresponding antigenic protein [6] and induce cytotoxic T lymphocyte activation and antibody production by presenting these antigens’ subunits on target cells’ surfaces [7].

However, severe concerns have been raised about the immunization status and safety of dialysis patients after vaccination due to their distinct clinical condition [8]. End stage renal disease (ESRD) patients are at high risk of developing severe COVID-19 complications due to the uremia-induced impaired immune system, the incapability of self-isolation [8,9,10], and the increased prevalence of comorbidities such as cardiovascular or chronic respiratory disease, diabetes, hypertension, malignancy, and liver failure. Previous studies have demonstrated the inadequate immunogenicity of other vaccine types, e.g., hepatitis B, influenza, diphtheria, and tetanus, with lower antibody titers and short-termed seroprotection in this at-risk group [11,12]. Furthermore, according to Glenn et al. [13], most clinical trials regarding COVID-19 vaccines have explicitly excluded patients with chronic kidney disease (CKD). Specifically, in Pfizer’s trial, renal patients accounted only for 0.7% of the randomized individuals, far below their distribution in the general population [14]. Consequently, further evidence is needed to thoroughly understand anti-SARS-CoV-2 vaccines’ impacts on hemodialysis patients (HDPs). Nevertheless, most studies contemplating vaccine efficacy in dialysis patients utilize mRNA-based vaccines, and, interestingly, studies comparing different types of COVID-19 vaccines in dialysis patients have concluded that mRNA-based vaccines are superior to other vaccines available for COVID-19 in terms of infection prevention and safety [15,16,17].

The aim of this review is to investigate the cellular and humoral immune response patterns induced in dialysis patients after administration of the BNT162b2 or mRNA-1273 vaccines.

## 2. Vaccine-Elicited Humoral Immune Response among Dialysis Patients

Table 1 includes studies assessing both the cellular and humoral responses elicited in dialysis patients after the administration of a two-dose vaccine regimen with either the BNT162b2 or mRNA-1273 vaccines and in whom no prior SARS-CoV-2 infection was reported. Table 2 includes studies assessing the added benefit of a third and/or fourth booster dose with an mRNA-based vaccine in HD patients already vaccinated with a two-dose mRNA-based vaccination regimen.

The adequate laboratory examination of humoral response levels plays a highly significant role in assessing vaccination efficacy. Serological protocols relying on the quantification of specific anti-viral antibodies are considered as reliable biomarkers [34]. As immunoglobulins targeting receptor binding domain (RBD) account for up to 90% of the formed anti-SARS-CoV-2-specific antibodies [35], all analyzed studies aim to measure IgG or IgA titers against the S1 domain. This subunit of spike viral protein mediates angiotensin converting enzyme-2 (ACE-2) host receptor recognition and viral protein binding, enabling the subsequent cell membrane fusion process [36,37].

### 2.1. Studies Assessing a Two-Dose mRNA-Based Vaccination Regimen on the Humoral Response in Dialysis Patients

Overall, case-control studies have demonstrated a defect in the humoral immunity elicited by HDPs compared to healthy participants after two doses of the mRNA vaccine, regardless of the diagnostic test used [21,22,23,24]. Espi et al. [21] screened 106 HDPs 10–14 days after the second dose of BNT162b2 vaccine and registered 19 non-responders (18%), in contrast to the control group in which 100% seroconversion was detected. In accordance with the above results, Strengert et al. [23] and Stumpf et al. [34] detected diminished antibody levels in patients undergoing HD, which, however, reached higher rates of positive immune responses of up to 95%. Of great interest were the findings of Schrezenmeier et al. [22] who observed a gradual increase in the proportion of cases reactive for SARS-CoV-2-IgG antibodies between 1- and 3-week samples, which finally seemed to decrease at a 10-month follow up. Regarding cohort studies, Broseta et al. [20] and Bertrand et al. [18] examined a total of 185 HDPs and detected viral-specific IgG titers at 95.4% and 88.9% of the screened population, 3 weeks and 1 month after the second injection, respectively. Remarkable discoveries were carried out by an investigation comparing humoral immunity induced in HDPs after BNT162b2 vaccination to physical viral infection. HDPs administered vaccination showed significantly weaker antibody production, noting a deviation of ≈30% in the responders’ rates compared to convalescent individuals, whose response was close to 90%. The augmented inflammatory response against encountered viral antigens in dialysis patients after natural SARS-CoV-2 infection, as opposed to the weaker vaccine-elicited immune response, was proposed as a possible explanation for the aforementioned findings [19]. Alternative, well-established methods that assess humoral immunogenicity were also exploited in other studies, including quantification of IgG targeting nucleocapsid protein subunit (NCP) [23,24] and evaluation of the neutralization capacity of vaccine-elicited immunoglobulins [19,22,23,24,25]. The latter reflects more accurately the patients’ immunological profiles by determining antibodies’ abilities to suppress viral growth [38]. This assay revealed that in HDPs, response alterations are also detected in antibodies’ functionality, leading to a reduced neutralizing potential of up to 94.7% and 77.78% of the population examined by Stumpf et al. [24] and Schrezenmeier et al. [22], respectively.

In conclusion, evidence from analyzed vaccine studies evaluating both the cellular and humoral responses indicates a significantly lower and delayed humoral response in HDPs after vaccination, compared to healthy individuals, that reached a peak in antibody titers 3–5 weeks after the second dose. 

Markedly impaired anti-BNT162b2 humoral responses were also observed by case-control studies evaluating only the antibody status of vaccinated patients. More specifically, according to Rincon-Arevalo et al. [39], a delayed but weak humoral immune response was observed in dialysis patients during measurement of viral-specific IgG and IgA, recording 70.5% and 68.2% positive responders, respectively, while in the control group, all individuals were seroconverted almost one week after the second dose. Additional follow-up samples 3–4 weeks post-vaccination showed that although the IgG titers increased substantially, IgA counts and surrogate neutralization markers remained stable. IgG detection assays were also exploited by Jahn et al. [40] and Grupper et al. [41] 2 weeks and 1 month after BNT162b2 vaccine administration. In the first study, healthy individuals registered median antibody levels of up to 800 AU/mL, and titers in HDPs reached a median of 366.5 AU/mL, while in the second study, corresponding measurements were up to 7.401 AU/mL and 2.900 AU/mL, respectively, for the two groups. In accordance with these findings, a study with age-matched controls observed that 18% of patients in the dialysis group did not reach the detection threshold for both RBD-specific and neutralizing antibodies, while all healthy controls exceeded the pre-determined threshold [42]. Yanay et al. [43] also came to relevant ascertainments, since patients in the HD group exhibited lower anti-spike antibody development and concurrently higher probability of a post-vaccination COVID-19 infection in contrast to the healthy population. 

### 2.2. Effect of a Third and/or Fourth Booster Vaccine Dose on the Humoral Response in Dialysis Patients

The suboptimal humoral response after a two-dose vaccine regimen and the need for continuous and robust antibody responses in dialysis patients necessitates modifications and/or enhancements to the primary vaccination regimen. Although vaccination with two doses of an mRNA-based vaccine prevents severe SARS-CoV-2 infection to a significant extent in dialysis patients, the waning humoral response that ensues about 4 months after the last dose increases the risk of reinfection without excluding the possibility of severe disease [44]. A booster vaccine dose has been indeed found to significantly augment the antibody response against SARS-CoV-2 in dialysis patients. Both BNT162b2 and mRNA-1273 vaccines have demonstrated promising findings in studies evaluating antibody response to a booster vaccine schedule. Verdier et al. [26] assessed 124 COVID-19-naïve HDPs with regard to humoral immune response, before and after a third dose of the BNT162b2 vaccine, by measuring the anti-S1 IgG titer, and they found a response rate of 82.9% and 95.8% before and after the third dose, respectively. In accordance with the above, Agur et al. [27] also examined the role of a third dose of the BNT162b2 vaccine in 80 HDPs and reported a seropositivity rate of 78% and 96% before and after the booster dose, respectively. In addition, anti-S1-RBD antibody titers, which were shown to diminish within 6 months of the second dose of the BNT162b2 vaccine, increased substantially following booster dose administration, while the difference in the mean antibody titers between HDPs and healthy controls decreased. Notably, this study reinforced previous observations stating that an extended dosing interval of at least 3 months takes full advantage of the booster vaccine’s properties [45], since following this extended-duration regimen, 76% of HDPs achieved a robust immune response, with anti-S1-RBD antibody titers exceeding 4.160 AU/mL. Regarding the effects of a third dose of the mRNA-1273 vaccine on the humoral response, Gallego-Valcarce et al. [29] studied 178 HDPs, 138 of which received the mRNA-1273 vaccine, with the remaining 40 receiving the BNT162b2 vaccine. All patients initially received a two-dose regimen with an mRNA-based vaccine. Results showed that 4 months after the booster dose, the humoral immune response, as determined by measuring the anti-SARS-CoV-2-S1-RBD antibody titer, had risen to 91.7% of HDPs, whereas the respective rate four months before the third dose was only 52.5%. Moving beyond the three-dose regimen, Becker et al. [28] assessed the effect of a fourth dose with the mRNA-1273 vaccine in 50 HDPs who had previously received three doses of the BNT162b2 vaccine. The ACE-2 binding inhibition activity was used a surrogate marker of virus neutralization and vaccine efficacy. While the decline of the humoral response was not as steep after the third as it was after the second dose, only 64% of HDPs remained above the ACE-2 binding inhibition threshold. Following administration of the fourth dose, the percentage of HDPs who surpassed the threshold rose to 96%. The above findings reinforce the importance of a booster vaccine dose in HDPs, since this practice leads to enhanced humoral response, sustained antibody levels capable of inducing neutralization, and overall protection from severe disease.

## 3. Vaccine-Elicited Cellular Immune Response among Dialysis Patients

### 3.1. Studies Assessing a Two-Dose mRNA-Based Vaccination Regimen on the Cellular Response in Dialysis Patients

Although antibodies are considered to play a significant preventive role against COVID-19 infection, T-cell effects also complement this protective purpose, with the BNT162b2 vaccine eliciting robust CD8+ and CD4+ RBD-specific T-cell responses [46]. A promising tool, and the most frequently used technique for evaluating the state of cellular immune activity, featured in many studies, is the interferon-gamma release assay (IGRA). Interferon-γ (IFNγ) is a pleiotropic cytokine crucial for host defense against viral and intracellular bacterial infections and is predominantly secreted by effectors of both the innate (natural killer cells, dendritic cells, and neutrophils) and the acquired immune system (CD4+ and CD8+ T lymphocytes) [47,48]. This molecule is responsible for immune cell recruitment and activation, and it also mediates the expression of IFNγ-regulated genes, with final derivatives including mediators of inflammation and apoptosis, and immune and transcriptional modulators [49,50,51]. IGRA can sensitively identify reactive T-cells to SARS-CoV-2 in blood samples [52] after overnight in-vitro stimulation with pathogen peptides [53].

In line with humoral immunogenicity, the T-cell-mediated IFNγ-response was significantly diminished in HDPs after completion of the recommended vaccination regimen, regardless of the days elapsed from the last injection. Schrezenmeier et al. [22] and Stumpf et al. [24] recorded 67.7% and 78% HDPs classified as reactive by IGRA, while corresponding proportions in control groups were up to 99.3% and 86%, respectively. The latter study also noted relevant immune patterns by applying deep immunophenotyping by fluorescence activating cell sorting (FACS) analysis. Massa et al. [54] noticed that the frequency of blood IFNγ-secreting cells increased from 7.0 cells per million peripheral blood mononuclear cells (PBMCs) 28 days after the second dose to 44.5, 28 days after the third dose (*p* < 0.0001). In addition to IFNγ responses, interleukin (IL)-2 levels also increased between the second and the third vaccine dose. Consistent findings were demonstrated by Strengert et al. [23], although none of the other 12 cytokines analyzed, except for IL-8 and chemokine (C–C motif) ligand-2 (CCL-2), differed significantly between healthy individuals and HDPs. Additionally, lower levels and diminished functionality of SARS-CoV-2-specific T-cells were detected in vaccinated people undergoing HD treatment compared to patients convalescing from physical infection, which could be attributed to a more potent antigenic challenge and lymphocyte recruitment in the latter group [38]. Nonetheless, a conflict has been raised regarding the correlation between mRNA vaccine-elicited humoral and cellular response in this cohort, with Espi et al. [21] recommending a significant correlation between the anti-RBD IgG titers and the levels of spike-specific CD4+ cells, while Broseta et al. [20] suggested that there was no correlation between these two parameters. Furthermore, CD8+ and CD4+ T-cell response patterns differed, with CD8+ T-cell measurements showing lower proportions of quantifiable results, though CD8+ status did not influence HDPs’ clinical and biological characteristics [21]. Inconsistent with data mentioned above, a study analyzing immunogenicity in 10 HDPs and 45 kidney transplant recipients (KTRs) one month after two doses of Pfizer’s vaccine, detected a positive cellular response in up to 100% and 57.8% of patients, respectively, suggesting the greatest efficacy of the BNT162b2 vaccine in the transplanted population [18]. Duni et al. [25] also studied the cellular immune response among HDPs and KTRs by assessing natural killer and natural killer T (NKT)-cells, which are considered to bridge the innate and acquired arms of the immune system, thus organizing the adaptive immune responses and immunoregulation. Regarding NKT-cell (CD3+ CD16+ CD56+) counts, an increase was observed in KTRs between the first and second dose, while HDPs displayed a different pattern of change in NKT-cell counts, where they increased substantially after the first dose, whereas a decrease was subsequently observed after the second dose, with their levels, however, remaining higher than baseline.

Generally, a considerable lack of information on cellular immunity in HDPs following completion of any type of available COVID-19 vaccine scheme was identified in the literature, highlighting the urgent need for further investigation. 

### 3.2. Effect of a Third and/or Fourth booster Vaccine Dose on the Cellular Response in Dialysis Patients

The cellular response after a booster vaccine dose has been less extensively analyzed compared to the vaccine-elicited humoral response, but results of associated studies provide insight into the potential protective role of T-cell-mediated immune responses against SARS-CoV-2 infection among dialysis patients. Melin et al. [30] analyzed the cellular response in 50 HDPs after a third dose of the BNT162b2 vaccine by measuring IFNγ production by T-cells incubated and stimulated with the COVID-19 spike protein. Of the 40 patients who were followed for the entire study, 55%, 85%, and 71% had a positive cellular response 7–15 weeks after the second dose, 3 weeks, and 3 months after the third dose, respectively. However, T-cell reactivity, although higher after the third dose, was not statistically significant when compared to the reactivity measured after the second dose (*p* = 0.96). Additionally, there was a statistically significant decline in the T-cell response between 3 weeks and 3 months after the third dose (*p* < 0.001). This observation needs to be interpreted cautiously, since it may reflect normal T-cell physiology rather than failure of the third dose to achieve a sustained cellular response. Becker et al. [28] assessed the cellular response after a three-dose regimen with the BNT162b2 vaccine and a subsequent fourth dose of the mRNA-1273 vaccine in 50 HDPs compared to 30 healthy controls. IFNγ was used as a marker of the cellular response. Overall, an incremental and stable cellular response was observed after the third and fourth dose of administration. The T-cell response after the third dose was comparable to the response observed after the second dose, but the difference was not statistically significant (*p* = 0.564). When comparing the T-cell response after the third and after the fourth dose, however, a statistically significant difference was observed (*p* < 0.0005). 

The results of the above studies highlight the need to conduct more research regarding the effect of booster dose administration on the T-cell response, since published results, although promising, are scarce, and thus definite conclusions are hard to make. 

## 4. Variables Affecting Immune Response in Dialysis Patients

Among the factors associated with poor immune responses after vaccination completion in HDPs, the presence of an immunosuppressive regimen was the most reported [18,20,21,23,24,55]. More specifically, patients undergoing immunosuppressive therapy tended to have less frequent seroconversion incidents accompanied with lower anti-spike IgG levels [20,23], while regarding cellular immunity, they almost never had detectable spike-specific CD4+ or CD8+ cells, according to Espi et al. [21]. The choice of immunosuppressive agent also influenced the results, as belatacept, mycophenolate mofetil (MMF), and calcineurin inhibitors seemed to cause higher response failure rates compared to glucocorticoids and mammalian target of rapamycin (mTOR)-inhibitors [18,24]. 

Other predictors of the observed weak immunogenicity among dialysis patients are associated with the clinical characteristics of the analyzed population. Patients with kidney failure are characterized by decreased lymphocyte count, systemic inflammation, malnutrition, and a defective clearance of uremic toxins, with all these parameters suggested to affect vaccination outcomes [22]. The uremic milieu in dialysis patients could potentially induce a detrimental effect in antibody development and regular function [21]. Furthermore, in most cases, older age was negatively associated with lower immunity [21,22,23,24]. Of particular interest, besides for the inadequate humoral protection in HDPs, is a case-control study revealing that age and gender influence immunogenicity patterns, with men producing lower immunoglobulin levels [44]. However, controversial data have emerged while evaluating the impact of age on humoral responses in the vaccinated population. Grupper et al. [41] and Simon et al. [42] established that age correlated inversely with antibody titers both in controls and HD patients, in contrast to Speer et al. [56], Rincon-Arevalo et al. [39], Jahn et al. [40], and Yanay et al. [43], who suggested that age-related differences were detected only in one of the two studied cohorts, either the control [42,43] or the HDPs [40,56]. Dialysis quality was also linked with more sufficient antibody protection in patients not treated with immunosuppressants [21]. Additional risk factors contributing to an extent to discrepancies evidenced between HDPs and healthy volunteers include time on dialysis, body mass index (BMI), sex, and comorbidities. In certain studies, cohort composition differed significantly regarding the control group and the dialysis population, with the latter counting more men, individuals with higher BMI, and multiple drug-requiring comorbidities, such as obesity, diabetes mellitus, cancer, liver cirrhosis, and cardiovascular disease, characteristics that may adversely influence the observed immune patterns [21,23,24]. Relevant recommendations were also made by Swai et al. [57], Carr et al. [58], Hou et al. [59], and Yen et al. [35], who further suggested that adverse immune responses could also be influenced by lower serum albumin levels [58], higher intravenous iron sucrose doses [58], insufficient vitamin D, and erythropoietin supplementation [59].

However, reviewed studies indicate that increasing the viral exposure of this vulnerable cohort may circumvent their immune dysfunctions, as previous COVID-19 infection was associated with favorable antibody responses [20,21]. In addition, a predominant predictor of both humoral and cellular response in HDPs and KTRs, but not in controls, was the type of mRNA vaccine used. In the dialysis group, the BNT162b2 vaccine achieved a 10% lower positive seroconversion rate and fewer positive cellular responders compared to the mRNA-1273 vaccine [24]. In agreement with these findings, Kaiser et al. [60] noted 2.98-fold higher anti-S-antibody titers in HD patients administered Moderna’s vaccine. To conclude, a considerable number of predictive factors has been recommended by the published literature, though their specific contribution in individuals’ immune responses to SARS-CoV-2 vaccines has yet to be elaborately investigated. 

## 5. Recommended Strategies to Augment the Immune Response to SARS-CoV-2 Vaccination in Dialysis Patients

An optimal approach to strengthen the immune response to SARS-CoV-2 vaccination in dialysis patients requires adherence to a vaccination regimen. This could be accomplished via a meaningful interaction between the healthcare provider and the patient and should aim to reinforce the positive outcomes following vaccination. Results from published studies support the administration of an mRNA-based vaccine in dialysis patients, both as part of a primary vaccine series and also during booster dose administration. Vaccines with a different mechanism of action have been shown to confer lower cellular and humoral immune response rates when compared to an mRNA-based vaccine, although it needs to be noted that studies contemplating a non-mRNA-based vaccine regimen in dialysis patients are limited. Regarding the optimal mRNA-vaccine that should be used, studies have found that the mRNA-1273 vaccine is associated with higher antibody titers when compared to the BNT162b2 vaccine, although antibody neutralization capacity and cellular responses between these two vaccines have not been studied extensively, making conclusions about the most appropriate mRNA-based vaccine in dialysis patients problematic [61,62]. As booster doses have been shown to accentuate the immune response to SARS-CoV-2 vaccination in dialysis patients, several aspects need to be taken into consideration. Time of booster dose administration since completion of the primary vaccine series seems to be important, since an extended dosing interval has been found to be more beneficial in augmenting the immune response by taking advantage of the booster vaccines’ properties [45]. Nevertheless, more studies are needed to examine the waning immunity after vaccine administration in dialysis patients and identify variables that affect it in order to more accurately reach a consensus regarding the optimal time of booster dose administration [63]. The number of additional booster doses is also of great concern, since many studies have found statistically significant improvements in the immune response to vaccination following multiple booster doses when compared to a single booster dose [28]. Laboratory confirmation of immune status following booster dose administration can potentially guide the need for future booster doses, with antibody neutralization capacity measured in most studies as the most reliable marker of successful protection against SARS-CoV-2. Finally, the aggressive control of risk factors that are negatively associated with the immune response to SARS-CoV-2 vaccination is deemed crucial, since the effective control of variables adversely correlating with vaccine efficacy can prevent suboptimal humoral and cellular immune responses from occurring (Table 3).

## 6. Conclusions

ESRD patients in HD treatment are shown to develop a promising but impaired humoral and cellular immune response, compared to the healthy population, after completion of the vaccination regimen with the available mRNA anti-SARS-CoV-2 vaccines, BNT162b2 and mRNA-1273. Taking into consideration the risk factors associated with this vulnerable group, adaptation of vaccination protocols should be a health priority. Booster dose administration with mRNA-based vaccines seems to augment both the humoral and cellular responses in DPs, and implementation of this strategy may result in sustained immunological responses in this high-risk group. Additionally, further research regarding the lifespan and the effectiveness of observed responses are urgently needed, with great attention to patients receiving immunosuppressive therapy, older individuals, and those with comorbidities, conditions deemed to adversely contribute to immunization outcomes.

## Figures and Tables

**Table 1:** Studies * evaluating the cellular and humoral immune responses after a 2-dose vaccination regimen with an mRNA-based vaccine in dialysis patients.

Author; Country	Study Design; Sample Size	Mean and/or Median Age (±SD) (yrs.)	Sex Males/Females	Vaccine Type; Number of Doses (Dosage Interval)	Timing of Blood Sampling Post Vaccination	Diagnostic Test; Criteria for Positive Response	Response Rate
						Humoral:	
						ARCHITECT IgG II	
Bertrand et al. [18]; France	Cohort; 45 KTRs, 10 HDPs	KTRs: 63.5 ± 16.3 HDPs: 71.2 ± 16.4	KTRs: 23/22; HDPs: NR	BNT162b2; 2 (21 days)	1 month	Quant test (Abbott); titers >50 AU/mL Cellular: Detection of SARS-CoV-2– reactive IFNγ-producing T-cells; SFC > 3 SDs of spot	KTRs: - Humoral: 17.8% - Cellular: 57.8% HDPs: - Humoral: 88.9% - Cellular: 100%
						numbers in wells	
						without antigens	
						Humoral: SARS-	
						CoV-2 IgG kit	Convalescent HDPs:
Blazquez-Navarro et al. [19]; Germany	Cohort; 18 Convalescent HDPs, 22 Vaccinated HDPs	Convalescent HDPs: 62 (39–78) Vaccinated HDPs: 67(43–80)	24/16	BNT162b2; 2 (NR)	Convalescent HDPs: median 60 days post vaccination (17–441); vaccinated HDPs: median 158 post infection (61–290)	(EUROIMMUN, Lübeck, Germany); NR Cellular: Cytokine expression by S-protein activated T	- Humoral: 85% < R < 90% - Cellular: ≈100% Vaccinated HDPs: - Humoral: - 50% < R < 60%
						cells (IFNγ, IL2,	- Cellular: 70% < R < 80%
						TNFα); NR	
						Humoral: Siemens	
						Healthineers Atellica	
						IM SARS-CoV-2 IgG	
						(sCOVG) assay; Anti-	
Broseta et al. [20]; Spain	Cohort; 175 HDPs	70.90 ± 14.96	118/57	100 HDPs: mRNA- 1273; 2 (28 days) 75 HDPs: BNT162b2; 2 (21 days)	3 weeks	S1-RBD IgG ≥ 1U/mL Cellular:Intracellular Cytokine Stimulation Assay; Flow cytometric detection > 10 events of CD4+ IFNγ+ CD69+,	Humoral: 95.4% Cellular: 62%
						with greater than 2-fold	
						change compared with	
						the unstimulated	
						condition	
						Humoral: Maglumi	
						SARS-CoV-2 S-RBD	HDPs: -Humoral: 82%
Espi et al. [21]; France	Case-Control; 106 HDPs, 30 Controls	HDPs: 64.9 ± 15.2 Controls: 46.6 ± 14.8	HDPs: 69/37 Controls: 14/16	BNT162b2; 2 (3–5 weeks)	10–14 days	IgG test (Snibe Diagnostic); titers > 1 AU Cellular: QuantiFERON SARS-	- -Cellular:62% (CD4+), 42% (CD8+) - Controls: - -Humoral: 100% - -Cellular: 100%
						CoV-2 test (Qiagen);	(CD4+), 70% (CD8+)
						NR	
						Humoral: anti-SARS-	DPs: -Humoral: 55.56% (1w), 88.9% (3w), 84.37% (10w) - -Cellular: 67.74% (3w) Controls: -Humoral: NR - -Cellular: 93.33% (3w)
						CoV-2-S1 ELISA	
		DPs: 74.0 (IQR				(EuroimmunMedizinisch	
Schrezenmeier et al. [22]; Germany	Case-Control; 36 DPs, 44 Controls	66.0, 82.0) Controls: 80.0 (IQR 75.75,	DPs 25/11 Controls: 14/30	BNT162b2; 2 (21 days)	≈3–4 and ≈10 weeks	eLabordiagnostika AG, Lübeck, Germany); OD > 1.1	
		82.25)				Cellular: interferon-	
						gamma release assay	
						(IGRA)	
						Humoral:	
						MULTICOV-AB;	DPs: -Humoral: 95%
Strengert et al. [23]; Germany	Case-Control; 81 HDPs, 34 Controls	HDPs: 69 (±18) Controls: 54.5 (±15)	HDPs: 47/34 Controls: 6/28	BNT162b2; 2 (21 days)	21 days	NR Cellular: SARS-CoV-2 Interferon Gamma Release Assay;	- -Cellular: 78% KTRs: -Humoral: 42% - -Cellular: 30% - Controls: -Humoral: 99%
						IFNγ concentrations	- -Cellular: 86%
						>200 mIU/mL	
						Humoral: SARS-CoV-2	
						specific IgG- or IgA-	
						antibody reactions	
						(S1,NCP,RBD)	
						(Euroimmun); de novo	
						antibody development	
						Cellular: SARS-CoV-	
						2 specific IFNγ	
Stumpf et al. [24]; Germany	Case-Control; 1256 DPs, 368 KTRs, 144 Controls	DPs: 67.6 ± 14; KTRs: 57.3 ± 13.7 Controls: 48 ± 11.9	DPs: 818/438; KTRs: 241/127 Controls: 34/110	1412: mRNA-1273;2 (28 days) 356: BNT162b2; 2 (21 days)	4–5 weeks	release assay (IGRA); results ≥ 100 mIU/mL and deep immunophenotyping by FACS analysis; CD4+ T-cells expressing CD154	HDPs: -seropositive: 91.8% - Antibody titers: 5759.9 ± 6771.6 - KTRtS:-seropositive 29.6% - Antibody titers: 113.9 ± 300.0
						and CD137 and	
						CD8+ T-cells	
						expressing CD137	
						with simultaneous	
						production of at least	
						one of	
						IL2/IL4/IFNγ/TNFα/	
						GrzB	
						Anti-SARS-CoV-2	HDPs: seropositive: 91.8% KTRs: seropositive 19.6% Antibody titers: HDPs > KTRs (5759.9 ± 6771 vs. 113.9 ± 300 g/dL)
						Antibody Response:	
						-Serologic response was	
Duni et al. [25]; Greece	Case Control; 34 HDPs, 54 KTR	HD:69.4; KTRs: 58.2	HD:23/11; KTRs: 38/54	BNT162b2; 2 (21 days)	21 days	assessed by using the ARCHITECT IgG II Quant test (Abbott)	
						-Flow Cytometry	
						Analysis	

* Studies (excluding the study conducted by Bertrand et al.) including dialysis patients diagnosed with prior or current SARS-CoV-2 infection were excluded from the table. Abbreviations: SARS-CoV-2: Severe Acute Respiratory Syndrome Coronavirus 2; mRNA: messenger ribonucleic acid; R: Response Rate; IQR: Interquartile Range; SD: Standard Deviation; HDPs: Hemodialysis Patients; PDPs: Peritoneal Dialysis Patients; KTR: Kidney Transplant Recipient; anti-S1-RBD: anti-Spike Protein S1 Receptor Binding Domain; IgG: Immunoglobulin G; IFNγ: Interferon gamma; anti-SARS-CoV-2-Nc-Abs: anti-SARS-CoV-2 Nucleocapsid Antibodies; SARS-CoV-2-NT-Abs: SARS-CoV-2 Neutralizing Antibodies; NR: No Results.

**Table 2:** Studies * evaluating the cellular and/or humoral immune response after booster administration of anti-SARS-CoV-2 mRNA vaccines in dialysis patients.

Author; Country	Study Design; Sample Size	Mean and/or Median Age (±SD) (yrs.)	Sex Males/Females	Vaccine Type; Number of Doses (Dosage Interval)	Timing of Blood Sampling Post Vaccination	Diagnostic Test; Criteria for Positive Response	Response Rate
Verdier et al. [26]; France	Retrospective observational; 142 HDPs	71.1/74.0	103/39	BNT162b2; 3 (21 days between doses 1 and 2, not clearly stated between doses 2 and 3)	Sample 1: 46 days after second dose Sample 2: 35 days after third dose	Humoral: Siemens Atellica IM anti-S1-RBD SARS-CoV-2 IgG assay (Siemens Healthcare GmbH, Erlangen, Germany); titers > 1.2 U/mL	Humoral: 82.9% after two doses, 95.8% after three doses
Agur et al. [27]; Israel	Prospective comparative; 80 HDPs 56 controls	HDPs: 72.61 ± 11.78 Controls: 69.3 ± 5.32	HDPs: 56/24 Controls: 27/27	BNT162b2; 3 (21 days between doses 1 and 2, at least 6 months between doses 2 and 3)	Sample 1: 21 days before third dose Sample 2: 21 days after third dose	Humoral: anti-S1-RBD SARS-CoV-2 IgG II Quant assay (Abbott); titers >50 AU/mL	Humoral: 78% after two doses, 96% after three doses
Becker et al. [28].; Germany	Prospective comparative; 50 HDPs 33 controls	HDPs: 69.5 (60–79) Controls: 42 (32–55)	HDPs: 31/19 Controls: 10/23	BNT162b; 3 (21 days between doses 1 and 2, 6 months between doses 2 and 3) mRNA-1273; 1 (4 months between doses 3 and 4)	Samples 1 and 2: 3 and 16 weeks after second dose Samples 3 and 4: 4 and 17 weeks after third dose Sample 5: 3 weeks after fourth dose	Humoral: MULTICOV-AB and RBDCoV-ACE2 assays (cut-offs not stated) Cellular: SARS-CoV-2 IFNγ Release Assay and IFNγ ELISA (Euroimmun); titers > 200 mIU/mL	Humoral: varied according to antibody measured and variant of concern studied, but incremental antibody response with each vaccine dose. Humoral: incremental cellular response after the third and fourth vaccine doses.
Gallego-Valcarce et al. [29]; Spain	Prospective observational; 178 HDPs	68.7 ± 14.5	113/65	40 HDPs: BNT162b2; 3 138 HDPs: mRNA- 1273; 3 (4 weeks between doses 1 and 2, 5 months between doses 2 and 3)	Every month starting from the first dose, and up to 4 months after the third dose	Humoral: anti-SARS-CoV-2 S1- RBD IgG assay (Abbott Laboratories, Chicago, Illinois); titers > 50 AU/mL	Humoral: 77.8% after two doses, 97% after three doses
						Humoral: anti-	
Melin et al. [30]; Sweden	Retrospective observational; 50 HDPs	69.4 ± 14.1	31/19	BNT162b2; 3 (4 weeks between doses 1 and 2, 6–8 months between doses 2 and 3)	Samples 1 and 2: 7–15 weeks and 6–8 months after dose 2 Samples 3 and 4: 3 weeks and 3 months after dose 3	SARS-CoV-2 S1- RBD IgG II Quant assay; titers >100 AU/mL and anti-SARS-CoV-2 anti-N IgG assay; titers > 1.4 S/COCellular: IFNγ level measurement	Humoral: 88% after two doses, 95% after three doses Cellular: 55% after two doses, 85% and 71% 3 weeks and 3 months after three doses, respectively
						(ELISPOT)	
Broseta et al. [31]; Spain	Prospective observational; 153 HDPs	72.12 ± 14.44	84/69	71 HDPs: BNT162b2; 3 82 HDPS: mRNA- 1273; 3 (dosage intervals not specified)	Two weeks after the third dose	Humoral: Siemens Healthineers Atellica IM SARS-CoV-2 IgG (sCOVG) assay; 1–150 U/mL: non- or weak responder, >150 U/mL: responder	Humoral: 97.4% seroconverted (> 1 U/mL) after the third dose (qualitative outcome only)
Quiroga et al. [32]; Spain	Prospective observational; 164 PDPs	62 ± 13	113/56	123 PDPs: mRNA- 1273; 3 or 4 (intervals not specified) 41 PDPs: BNT162b2; 3 or 4 (intervals not specified)	28 days, 3 months, and 6 months after two doses, 6 and 12 months after three doses, 12 months after four doses	Humoral: CLIA, COVID-19 Spike Quantitative Virclia IgG Monotest (Vircell SL, Grenada, Spain); titers > 36 IU/mL	Humoral: 80% 6 months after 2 doses, 97% 12 months after 3 doses, 100% 12 months after 4 doses (not statistically significant to 3-dose schedule)
						Humoral: Elecsys anti-	Humoral: 90% seroconversion after 2 doses (50% after adjustment for anti-SARS-CoV-2-S-Ab levels using surrogate neutralization test), 100% after 3 doses. 97.3% HDPs with adequate neutralizing Abs after three doses
						SARS-CoV-2-Nc-Abs and	
						anti-SARS-CoV-2-S-Ab	
						(Roche Diagnostics,	
Kohmer et al. [33]; Germany	Prospective observational; 194 DPs (167 HDPs, 27 PDPs)	DPs: 69.6 ± 14.2 (HDPs: 71.0 ± 13.9, PDPs: 61.6 ± 13.8)	DPs: 110/84 (HDPs: 93/74, PDPs: 17/10)	BNT162b2; 2 (intervals not specified) mRNA-1273; 1 (6 months after second dose)	4 and 10–12 weeks after two doses, 4 weeks after third dose	Mannheim, Germany); titers > 0.8 U/mL. Measurement of SARS-CoV-2-NT-Abs with the ELISA-based GenScript SARS-CoV-2 Surrogate	
						Virus Neutralization Test	
						Kit (GenScript Biotech, NJ,	
						USA); inhibition > 30%	
						was considered positive	

* Studies including dialysis patients diagnosed with prior or current SARS-CoV-2 infection were excluded from the table. Only studies assessing dialysis patients who received a two-dose vaccination regimen with an mRNA-based vaccine prior to booster dose administration were included in the table. Abbreviations: SARS-CoV-2: Severe Acute Respiratory Syndrome Coronavirus 2; mRNA: messenger ribonucleic acid; SD: Standard Deviation; HDPs: Hemodialysis Patients; PDPs: Peritoneal Dialysis Patients; anti-S1-RBD: anti-Spike Protein S1 Receptor Binding Domain; IgG: Immunoglobulin G; IFNγ: Interferon gamma; anti-SARS-CoV-2-Nc-Abs: anti-SARS-CoV-2 Nucleocapsid Antibodies; SARS-CoV-2-NT-Abs: SARS-CoV-2 Neutralizing Antibodies.

**Table 3:** Recommended strategies to augment the immune response to SARS-CoV-2 vaccination in dialysis patients.

Recommended Strategy	Comments/Notable Considerations
Reinforce importance of vaccination in dialysis patients, ensure vaccine availability in dialysis patients, promote patient–physician relationships to establish goals and promote the benefits of vaccination	Although not directly associated with augmentation of the immune response, these measures are deemed crucial to increase vaccination rates in dialysis patients.
Primary vaccination series with an mRNA-based vaccine (either mRNA-1273 or BNT162b2)	Most studies report greater immune response outcomes with an mRNA-based vaccine when compared to other vaccine types in dialysis patients. Although the mRNA-1273 has been shown to confer higher antibody titers in dialysis patients compared to the BNT162b2 vaccine, more studies are needed to assess whether recommendations about a specific mRNA-based vaccine are justified. Currently, either vaccine can be recommended, and factors such as local availability as well as potential adverse effects from a specific vaccine need to be taken into account.
Booster dose administration with an mRNA-based vaccine	Booster doses significantly accentuate the immune response to vaccination in dialysis patients. Most studies have assessed an mRNA-based booster dose regimen. The dosing interval between the booster dose and last dose of primary vaccine series, intervals between booster doses, and the number of booster doses administered are factors that need to be assessed in future studies.
Periodic laboratory monitoring of the humoral and cellular responses to vaccination in dialysis patients	Although not directly associated with augmentation of the immune response following vaccination, monitoring of antibody titers, antibody neutralization capacity, and cellular response after vaccination can identify patients at risk that require additional booster doses due to the inevitable waning of immunity that ensues following vaccination, especially in dialysis patients.
Aggressive control of risk factors that negatively impact vaccine efficacy	It is crucial to eliminate or at least minimize the impact of well-reported risk factors that negatively correlate with immune responses after vaccination. Some variables, however, such as the use of an immunosuppressive regimen in kidney transplant patients, are hard to address, and, in such cases, careful monitoring and management of other risk factors may prove beneficial.
Inclusion of dialysis patients in trials exploring vaccine efficacy and safety	Studies that contemplate vaccine outcomes and safety in dialysis patients are needed to learn more about the immune response alterations in this population with regards to SARS-CoV-2 vaccination. This could solidify an appropriate vaccine strategy that is safe and effective.

Abbreviations: SARS-CoV-2: Severe Acute Respiratory Syndrome Coronavirus 2; mRNA: messenger ribonucleic acid.

## Data Availability

Not applicable.
